# Prevalence of Capgras syndrome in Alzheimer’s patients: A systematic review and meta-analysis

**DOI:** 10.1590/1980-57642018dn13-040014

**Published:** 2019

**Authors:** Gabriela Caparica Muniz Pereira, Gustavo Carvalho de Oliveira

**Affiliations:** 1 Acadêmica de Medicina do Centro Universitário de Brasília - UniCeub, DF, Brazil.; 2 Professor Adjunto do Centro Universitário de Brasília - Uniceub, DF, Brazil.

**Keywords:** Capgras syndrome, Alzheimer’s disease, dementia, delusion, meta-analysis, síndrome de Capgras, doença de Alzheimer, demência, delírio, metanálise

## Abstract

The association between Capgras syndrome and Alzheimer’s disease has been reported in several studies, but its prevalence varies considerably in the literature, making it difficult to measure and manage this condition.

**Objective:**

This study aims to estimate the prevalence of Capgras syndrome in patients with Alzheimer’s disease through a systematic review, and to review etiological and pathophysiological aspects related to the syndrome.

**Methods:**

A systematic review was conducted using the Medline, ISI, Cochrane, Scielo, Lilacs, and Embase databases. Two independent researchers carried out study selection, data extraction, and qualitative analysis by strictly following the same methodology. Disagreements were resolved by consensus. The meta-analysis was performed using the random effect model.

**Results:**

40 studies were identified, 8 of which were included in the present review. Overall, a total of 1,977 patients with Alzheimer’s disease were analyzed, and the prevalence of Capgras syndrome in this group was 6% (CI: 95% I² 54% 4.0-8.0).

**Conclusion:**

The study found a significant prevalence of Capgras syndrome in patients with Alzheimer’s disease. These findings point to the need for more studies on the topic to improve the management of these patients.

## RESUMO

A associação da síndrome de Capgras com a doença de Alzheimer é relatada em diversos estudos, porém a sua prevalência varia consideravelmente entre bibliografias, dificultando a real mensuração e manejo desta patologia.

**Objetivo:**

O objetivo deste estudo foi estimar, através de uma revisão sistemática, a prevalência da síndrome de Capgras em pacientes com a doença de Alzheimer, bem como revisar os aspectos etiológicos e fisiopatológicos relacionados à síndrome.

**Métodos:**

Uma revisão sistemática foi realizada utilizando-se as seguintes bases de dados: Medline, ISI, Cochrane, Scielo, Lilacs e Embase. A seleção dos estudos, extração de dados e análise qualitativa foi feita por dois pesquisadores de forma independente e seguindo rigorosamente a mesma metodologia, sendo as discordâncias resolvidas por consenso. A metanálise foi calculada utilizando-se modelo de efeito randômico.

**Resultados:**

Foram recuperados 40 estudos, dos quais 8 foram incluídos na presente revisão. Considerando todos os estudos incluídos, 1977 pacientes com doença de Alzheimer foram analisados, sendo encontrada a prevalência da síndrome de Capgras dentre esses pacientes de 6% (IC: 95% I² 54% 4,0-8,0).

**Conclusão:**

A presente revisão encontrou uma prevalência significativa da síndrome de Capgras nos pacientes com doença de Alzheimer. Tal achado implica na necessidade de mais estudos visando melhorar o manejo desses pacientes.

The aging population is a phenomenon accompanied by critical epidemiological changes, such as a higher incidence of chronic diseases. Among these conditions, dementias have the most significant impact on the elderly. According to data from Alzheimer’s Disease International, every three seconds, someone develops dementia in the world. There are an estimated 46.8 million people worldwide living with the illness, and its leading cause is Alzheimer’s Disease (AD).^1^

One of the signs that may be present in AD is psychosis, occurring in 42% to 84% of cases.^2^ The main symptoms are delusions, hallucinations, and Delusional Misidentification Syndromes (DMS), with a predominance of 30% in patients with AD.^3^ Among the DMS, Capgras syndrome is the most commonly reported, but its prevalence varies significantly depending on the study, ranging from 5%4 to 16%.^3^^,^ 4

Capgras syndrome (CS) was first described by French psychiatrist Joseph Capgras under the term “illusion of doubles” in 1923.^5^ In his first study on the subject, Capgras reported the case of patient M, a 53-year-old woman, without a previous history of psychiatric disorders. However, she began to be delusional after the death of her twin sons. Eventually, she started to believe that her husband and children, as well as herself, were all duplicates. Although the syndrome describes the replacement of people, some authors extend the concept to objects and pets. Henceforth, the delusion that causes patients to believe that a person, usually a loved one, has been replaced by a double has become known by the eponym “Capgras syndrome”. CS is a syndrome within the scope of Delusional Misidentification Syndromes.^6^

Initially, the etiology of CS seemed to be necessarily linked to psychotic episodes. There are reports of several cases of the syndrome associated with organic pathologies over the years, such as myxedema,^7^^,^ 8 encephalitis,^9^ multiple sclerosis,^10^ and lithium intoxication.^11^ In psychotic disorders, Capgras syndrome is still more common today in patients with schizophrenia and degenerative dementias. These pathologies account for 81% of all cases of CS.^12^

There is no consensus on theories of pathophysiology regarding CS. Also, these theories vary according to the time they were written. When J. Capgras described this pathology, it seemed to be fundamentally associated with psychotic episodes.^13^ Hence, psychodynamic theories were widely used to explain the pathophysiology of this disorder, supporting inferences, such as incestuous desires, suppressed homosexuality, and even the most broadly accepted psychodynamic theory claiming the symptoms of the syndrome are a response to ambivalent and repressed feelings that can subsequently be channeled against the ‘impostor’.

Also, organic theories were postulated, and CS became the basis for the creation of models using face recognition systems, such as the studies of Ellis and Young from 1990 onwards.^14^ Since then, several researchers have proposed models based on this syndrome, which strongly suggested that face recognition occurs through two pathways: the central and extended nervous systems. The central nervous system analyzes facial features. There are several important structures involved, such as the inferior occipital gyrus, the lateral occipitotemporal gyrus, and the superior temporal sulcus. The extended system collects emotional information related to the face in question, and is subdivided into two parts. One part is responsible for retrieving emotional information about the face, recruiting the anterior paracingulate cortex, posterior temporoparietal junction, anterior temporal cortex, and posterior cingulate cortex. The other part is responsible for analyzing emotional representations related to the face and involves the amygdala, insula, and striatum reward system.

Thus, taking this system as a basis, CS can be characterized as a breakdown of communication between the central and the extended nervous systems.^15^ Due to the high prevalence of DMS, and more specifically, CS, it is crucial to identify such syndromes and improve the treatment of patients with these disorders. Therefore, we took into account several aspects, such as data on the variation in prevalence of CS among patients with AD, and the lack of previous systematic reviews that address this theme. This study aims to verify the prevalence of CS in Alzheimer’s disease and review the scientific literature regarding the etiology, pathophysiology, clinical presentation, and treatment of this critical delusion. 

## METHODS

This study is a systematic review in which objective criteria were used for data collection as described below. Two independent researchers verified all data strictly following the same methodology.

### Registration

This review was registered in the International Prospective Register of Systematic Reviews (PROSPERO) under protocol: CRD42018103929.

### Data collection

We searched the MEDLINE, ISI, COCHRANE SCIELO, LILACS and EMBASE databases for papers about Capgras syndrome prevalence in patients with Alzheimer’s disease, using the following keywords: “Capgras syndrome” and “Alzheimer”. The corresponding terms in Portuguese were used in the LILACS and SCIELO databases.

### Search strategy

In the MEDLINE database, the search strategy used was: (“capgras syndrome” [MeSH Terms] OR (“capgras” [All Fields] AND “syndrome” [All Fields]) OR “capgras syndrome” [All Fields] ) AND (“Alzheimer’s disease” [MeSH Terms] OR (“Alzheimer’s” [All Fields] AND “disease” [All Fields] OR “Alzheimer’s disease” [All Fields] OR “Alzheimer’s” [All Fields]). The same strategy was adopted in other databases.

### Selection

The papers were selected based on their titles and abstracts. Subsequently, the items that matched the field of interest were read in their entirety. Original findings from studies evaluating the prevalence of CS in AD in English and Portuguese published after the search dates were also included. Papers about other DMS and not Capgras syndrome, papers on dementias other than AD, letters to the editor, publications other than reviews or non-original papers, single case reports or studies with the same samples, and papers without full access to the content were excluded.

### Data classification and tabulation

The following variables were evaluated for the selected studies: year of publication, sample size, and characteristics, prevalence of CS in AD, diagnostic tools for AD, CS identification instrument, and study location.

The variables for the selected studies were then compiled into a table (Table 1).

**Table 1:** Characteristics of studies included in the review.

Authors	Year of publication	Sample (n total)	Diagnosis criteria for Alzheimer's disease (AD)	Diagnosis criteria for Capgras syndrome (CS)	Prevalence of CS in AD	Country of study
Kwak et al.^18^	2012	230	NINCDS-ADRDA	Delusion Subscale of the Korean Neuropsychiatric inventory	4.3%	South Korea
Harciarek and Kertesz^19^	2008	392	NINCDS-ADRDA	Clinical interview with caregivers	5.9%	---
Josephs^20^	2007	47*	---	Explicit description in medical records	---	The United States
Mizrahi et al.^21^	2006	771	NINCDS-ADRDA	Dementia pyschosis scale	4.4%	Argentina
Harwood et al.^22^	1999	158	NINCDS-ADRDA	BEHAVE-AD	10%	The United States
Migliorelli et al.^23^	1995	103	NINCDS-ADRDA	Dementia pyschosis scale	5.8%	Argentina
Förstl et al.^3^	1994	50 and 56**	NINCDS-ADRDA	Clinical interview with caregivers	16%	England/Germany
Mendez et al.^4^	1992	217	---	Caregiver report	5%	The United States

NINCDS-ADRDA: National Institute of Neurological and Communicative Disorder and Stroke - Alzheimer's Disease and Related Disorders Association.

* Joseph's study initially used patients diagnosed with CS as sample.

** Förstl's study carried out a review o two studies with diferent samples.

### Qualitative analysis

The critical analysis proposed by Loney et al.,^16^ with adaptations, based on the analysis of 8 criteria, was used for this qualitative analysis, namely: random or total sampling; sample data source; sample size; research bias; measuring tools; response rate and refusal report; confidence interval and description of the study objective. Each criterion is worth one point. Studies with a score of 0 to 3 were considered low quality, 4 to 6 intermediate quality, and 7 to 8 high quality. The qualitative score was not an exclusion criterion.

### Quantitative analysis

The primary outcome was the prevalence of CS in patients with AD. Firstly, the prevalence was calculated for each selected study, with a confidence interval of 95%. These prevalences and their intervals were plotted on a single graph to compare both prevalence and confidence intervals.

The meta-analysis was calculated using random effect and the heterogeneity was assessed by the Chi-square test, with p<0.10 indicating significance, according to guidelines for this type of study design,^17^ and magnitude was calculated by I squared. The data were then plotted using a Forest Plot.

Due to the small number of studies included in the meta-analysis, no meta-regression was performed, in accordance with literature guidelines on the minimum number of studies for this method.^17^

## RESULTS

The software R version 3.4.1 was used for data analysis and graphs.

**Figure 1:**
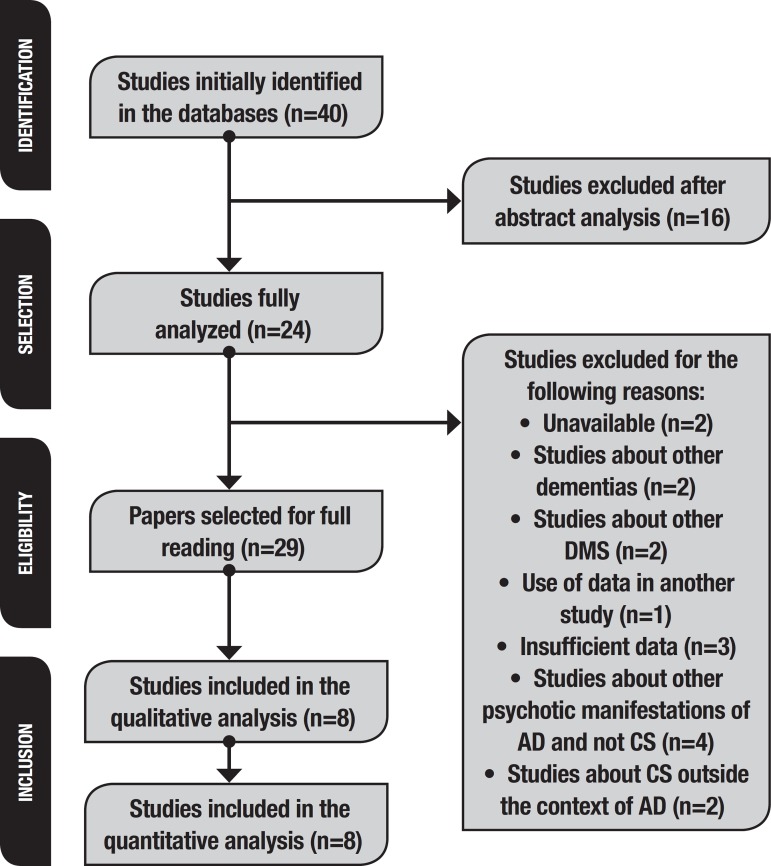
Study selection and inclusion flowchart.

Initially, 40 studies were retrieved (37 via Medline, and three via Lilacs). However, after reading of titles and abstracts, 16 were excluded (3 due to language - 2 in French and 1 in German - and 13 because the methodology did not meet the selection criteria described). The remaining 24 studies were read in full, of which 16 were rejected after applying the exclusion criteria. Finally, 8 articles were included in this review. The flowchart representing the study retrieval process is shown in Figure 1. 

Of the selected studies, only three were not cross-sectional: one of the samples used in the study of Forstl et al.,^3^ the longitudinal retrospective study by Mendez et al.,^4^ and the retrospective study by Josephs.^20^ All studies used patients treated at specialized clinics as samples. The findings were published between 1992 and 2012 in South Korea, England, Argentina, the United States, and Germany. The NINCDS/ADRDA (National Institute of Neurological and Communicative Disorders and Stroke/Alzheimer’s Disease and Related Disorders Association) criteria were used as diagnostic criteria for AD. The studies varied considerably in terms of sample size, and especially in the method used to diagnose Capgras Syndrome, as seen below.

The study by Kwak et al.^18^ was conducted in South Korea with 230 first-time AD treatment patients to compare the types of delusions present in Alzheimer’s patients. The sample was selected from recently diagnosed patients of an Alzheimer’s geriatric hospital. Using the NINCDS-ADRDA criteria, a multidisciplinary team evaluated the selected patients. The presence of delusions was determined by interviewing caregivers and using the K-NPI (Korean Neuropsychiatric Inventory) as a research tool. Patients were further analyzed for the severity of dementia using the Korean version of the Mini-Mental State Examination (K-MMSE), Clinical Dementia Rating scale (CDR), and Clinical Dementia Rating Scale-Boxes (CDR-SB). The overall prevalence of CS found among all patients in the study was 4%. Considering only the patients who presented some delirium, the prevalence of CS was 15.9%.

Harciareck and Kertesz’s^19^ study included 392 patients diagnosed with AD, among other degenerative diseases, using the NINCDS-ADRDA criteria. The study focused on the prevalence of DMS in neurodegenerative diseases. A multidisciplinary team analyzed the selected patients and verified the presence of DMS through direct questions to the caregivers and patients. Patients’ cognitive status, as well as their cognitive decline, was assessed using the Mini-Mental State Examination (MMSE), and the Dementia Rating Scale (DRS), respectively. The prevalence of CS among AD patients in the study was 5.9%. The prevalence of CS among patients with any DMS was 37%.

Josephs’^20^ study differed from the others. Firstly, because it was a retrospective study, and also, considering the primary sample of patients with CS and from these patients outlining which major underlying pathologies were associated. The sample was taken from the analysis of the medical records of patients treated at the Mayo Clinic from 1996 to 2006. The criterion used for the diagnosis of CS through the analysis of records was the explicitness of the syndrome in medical records. The prevalence of AD found among patients with CS was 14.9%, due to the diversity of the initial sample, this study was not included in the meta-analysis.

The study by Mizrahi et al.^21^ was conducted in Buenos Aires and included 771 patients treated at a clinic for patients with dementia diagnosed with AD using the NINCDS-ADRDA criteria. The focus of the study was to analyze clinical aspects related to the presence of delusions in patients with AD. Subsequently, a multidisciplinary team evaluated the patients and screened them for the existence of psychotic symptoms, including CS, which was determined by applying the Dementia Psychosis Scale (DPS) with caregivers. The prevalence of CS among AD patients in the study was 4.8%, rising to 13% when only patients with a type of delusion were analyzed.

The study by Harwood et al.^22^ was conducted in Miami in patients diagnosed with AD using the NINCDS-ADRDA criteria and who had already been seen at a specialized clinic, focusing on the clinical aspects of Alzheimer’s patients who manifested CS. Patients were evaluated by a multidisciplinary team to determine their cognitive function using the MMSE, functional deficits using the Blessed Dementia Scale, as well as the presence of behavioral disorders. The presence of CS was assessed by applying the Behavioral Pathology in Alzheimer’s Disease Rating Scale (BEHAVE-AD) with caregivers. The prevalence of CS in AD patients in the study was 10.1%. 

The study by Migliorelli et al.^23^ was conducted in Buenos Aires in 103 patients diagnosed with AD by the NINCDS-ADRDA criteria at a neurological clinic. A psychiatrist evaluated the patients, and several instruments were used to assess the presence of delusions, as well as other psychiatric symptoms. The Dementia Psychosis Scale, applied to patients and caregivers, determined the presence of CS. The prevalence of CS in patients with AD was 5.8%. Among patients with delusions, this prevalence was 29%.

The study by Forstl et al.^3^ differs from the others in that it is a summary of two other previously conducted studies. The first of these is a longitudinal study, in which patients with AD were followed. The evidence of psychiatric disorders diagnosed was collected by a retrospective interview with caregivers, and the diagnosis of dementia was subsequently confirmed post-mortem. The second study is a prospective study in which patients with AD, diagnosed by the NINCDS-ADRDA criteria, were followed to determine the appearance of psychotic symptoms. In both studies, the presence of CS was investigated through interviews with caregivers. In the first study, the prevalence of CS among patients with AD and delusions was 16%. In the second study, the prevalence of CS among all patients was also 16%, whereas the rate among patients with delusions was 25.8%.

The study by Mendez et al.^4^ is a retrospective study aimed at people recognition disorders, such as prosopagnosia, false identification of their image, CS, and transient misidentification. The medical records of 217 patients seen at a specialized Alzheimer’s clinic in Cleveland and who were diagnosed with AD and other psychiatric symptoms were reviewed. The diagnosis of CS was established through interviews with caregivers. The prevalence of CS among patients with AD and any psychiatric symptoms in the study was 5%. Among the people recognition disorders, CS prevalence was 20%.

**Figure 2:**
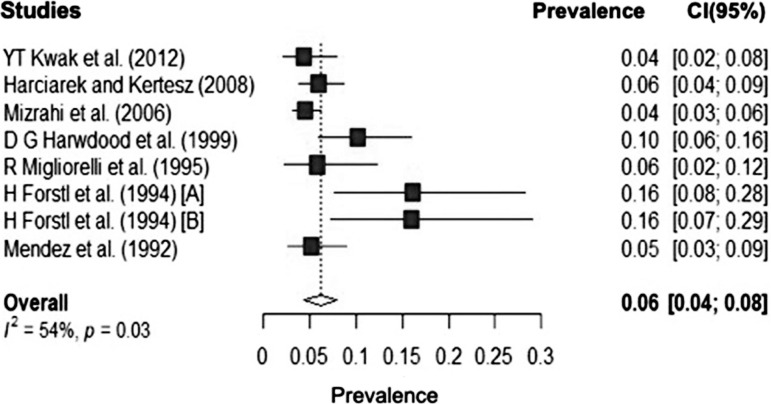
Prevalence of Capgras Syndrome in patients with Alzheimer’s disease.

The meta-analysis showed the prevalence of CS in the 1,977 AD patients analyzed as 6% (CI: 95% I² 54% 4.0-8.0) (Figure 2).

The heterogeneity found is considered moderate, taking into account I squared. However, because the number of studies in the review was less than 10, it was not possible to perform meta-regression to test variables.^17^

## DISCUSSION

The analysis of the prevalence of CS found among Alzheimer’s patients shows that this type of psychotic symptom is relevant in this population. Although it was not possible to test the variables for the heterogeneity found, some hypotheses can be formulated to explain it: different sample sizes, study design, demographic diversity, and systematized diagnostic instrument for CS. The selected and compared studies varied significantly concerning sample size, with the smallest sample being 50 patients in the study by Förstl et al.^3^ and the largest in the study by Mizrahi et al.^21^ with a sample of 771 patients. The analysis of the studies revealed the absence of investigations involving large sample sizes.

Also, the lack of extensive longitudinal studies is a factor to be considered, since psychotic manifestations in AD may appear at different times during the disease course. Therefore, follow-up of these patients is necessary to better understand and measure these symptoms.

Demographic difference is another point to be considered, as studies from various parts of the world, such as North America, South America, Europe, and Asia were included in this study. This diversity is essential, in the case of mental illnesses, as illustrated in the study by Kwak et al.^18^ There is a cultural difficulty in seeking medical help for older people with dementia and consequent underdiagnoses of Alzheimer’s and CS.

The diversity of instruments used to diagnose delusions and CS seems to have contributed to a variation in test results. The study with the highest difference in prevalence did not use a systematized instrument to diagnose CS. This finding points to the need for creating a standard system for syndrome diagnoses, besides tools that optimize this process.

Studies have shown that the main psychotic symptoms affecting AD patients are delusions, followed by DMS, of which CS is the most common. Among the comparative studies, the profile of AD patients most susceptible to developing CS was not mapped. However, there seems to be no gender difference.^22^ Regarding delusions, the most impacted person is the primary caregiver.^4^^,^ 19 ^,^ 22 Studies also show that the presence of CS in AD patients indicates a propensity for other symptoms, such as depression,^21^ anosognosia,^21^^,^ 23 and other delusions.^4^^,^ 19 ^,^ 22

Studies differ regarding prognosis. However, it is known that delusions, and consequently CS, are associated with a more significant cognitive deterioration of the patient. It is a fact that patients with CS have worse performance on scales, such as the MMSE,^18^^,^ 19 ^,^ 22 as well as its onset in moderate and advanced stages of the disease.^3^^,^ 18 ^,^ 19 ^,^ 22 ^,^ 23

Besides the epidemiological impact of CS in terms of AD, it is necessary to take into account the social effect. This concerns caregivers and the support network of the elderly, as some studies indicate that delusion in Alzheimer’s patients is a factor that may lead to abandonment.^21^^,^ 24

The present study has some limitations, such as the small number of quality studies on the subject, the difficulty mapping the profile of the patients most susceptible to developing CS, and the impossibility of comparing previous reviews, since no systematic reviews on the topic were found.

In conclusion, Capgras syndrome in patients with Alzheimer’s disease has a significant prevalence. It is relevant to consider this fact in this specific patient group. Further studies involving larger samples, as well as a longitudinal design, will be necessary to map the profile of AD patients more susceptible to developing CS. Another point to be considered is the need for development of a standard instrument to identify more cases.

Moreover, the treatment of the syndrome needs further investigation, where lack of studies makes managing these patients a challenge. Correct early identification of the syndrome will improve both the clinical management and quality of life of patients with Alzheimer’s. Further studies on CS and AD are also needed addressing the aspects of clinical/propaedeutic management.
